# Solid state chemistry for developing better metal-ion batteries

**DOI:** 10.1038/s41467-020-18736-7

**Published:** 2020-10-02

**Authors:** Artem M. Abakumov, Stanislav S. Fedotov, Evgeny V. Antipov, Jean-Marie Tarascon

**Affiliations:** 1 Skoltech Center for Energy Science and Technology, Skolkovo Institute of Science and Technology, Moscow, Russia 121205; 2 Department of Chemistry, Lomonosov Moscow State University, Moscow, Russia 119991; 3 Chimie du Solide-Energie, UMR 8260, Collège de France, 75231 Paris Cedex 05, France

**Keywords:** Batteries, Solid-state chemistry, Batteries, Batteries

## Abstract

Metal-ion batteries are key enablers in today’s transition from fossil fuels to renewable energy for a better planet with ingeniously designed materials being the technology driver. A central question remains how to wisely manipulate atoms to build attractive structural frameworks of better electrodes and electrolytes for the next generation of batteries. This review explains the underlying chemical principles and discusses progresses made in the rational design of electrodes/solid electrolytes by thoroughly exploiting the interplay between composition, crystal structure and electrochemical properties. We highlight the crucial role of advanced diffraction, imaging and spectroscopic characterization techniques coupled with solid state chemistry approaches for improving functionality of battery materials opening emergent directions for further studies.

## Abstract (web summary)

The development of high performing metal-ion batteries require guidelines to build improved electrodes and electrolytes. Here, the authors review the current state-of-the-art in the rational design of battery materials by exploiting the interplay between composition, crystal structure and electrochemical properties.

## Introduction

Invention of new functional materials is vital for the advancement in technologies that will move society towards high global standards of living. Electrode materials have played a crucial role in the development of highly performing Li-ion batteries, as was recognized by the 2019 Nobel Prize recompensing solid-state chemists for their decisive impact^1^. Yet, the vast number of compositions potentially available from the Periodic Table poses an overwhelming challenge for the materials science community to find new battery electrodes. Obviously, researchers desperately need solid guidelines while searching through this huge parameter space for the best chemical combinations and structures. Solid state chemistry is the art of building the desired atomic arrangements based on information hidden in the Periodic Table. Over the decades, this research field has evolved from the trial and error Edison’s approach to become a fully-fledged science delivering an unprecedented control over material’s structure and properties. This allowed building predictive models and conferring specific functional properties to a material, with an extra degree of freedom offered by defects in solids, structure dimensionality, and nanosizing. Nowadays, the context of research is displaced towards accelerated materials discovery and novel eco-compatible processes together with engineering advances for device fabrication and prototyping. Thus, solid state chemistry is still expanding pursuing our demands of understanding matter and transforming it to useful solids for emerging technologies.

**Fig. 1:**
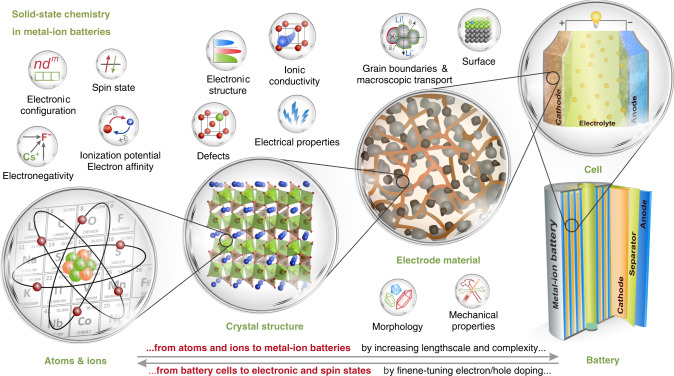
The “composition-structure-property” triad in metal-ion batteries. The individual properties of atoms and ions encoded in the Periodic Table determine the basic redox chemistry, which is fine-tuned by embedding into a certain crystal lattice, in which the peculiar electronic structure and defects define the operating potential, electrochemical capacity, electrochemical stability window, electronic and ionic conductivity. Situated at the next level of complexity, the electrode material combines surface modifications, morphology tuning, and control of grain boundaries optimized for high-energy density, rate capability, and cycling stability through advanced synthesis methods. Further step towards the electrode requires extensive engineering aimed at selecting proper conductive additives and binders and mastering the deposition techniques of the electrode slurry onto current collector. Note that the start and end points of this chain can be reversed, i.e. instead of playing with electronic and crystal structures to design better batteries, one could use the battery as an electrochemical reactor for fine tuning the chemical composition and electronic structure and preparing metastable compounds with unusual oxidation states.

Within the metal-ion battery technology the electrode reactions are based on reversible insertion/deinsertion of the alkali (or alkali-earth) cations A^+^ into the host electrode material with a concomitant addition/removal of electrons. In light of this ion-electron duality, it pertains to design an inorganic framework with the proper crystal and electronic structure to reversibly accept and release ions and electrons. This is the favored playground for solid-state chemists willing to better predict and improve battery functionality, as picturized in Fig. 1 with our personal perception of the “composition-structure-property” triad in the context of electrode materials. The specific energy of an insertion material (Wh kg^−1^ or Wh l^−1^) is defined as the product of the operating potential (V) and the capacity (Ah kg^−1^ or Ah l^−1^), both related to the crystal and electronic structure. Extraction of one A^+^ alkali cation is accompanied by loss of one electron which produces both a negatively-charged cationic vacancy and a positively-charged electronic hole in the M–L framework (M—transition metal, L—anion). Generating holes upon battery charge should be considered in the band structure context, but for a chemist it is tempting to link these holes to specific redox centers. Although strongly simplified, this concept enables solid-state chemists to rationalize the electrochemical behavior of electroactive materials on the basis of crystal chemistry, molecular orbitals, ionocovalency, bond valence approach, thermodynamics, and defect chemistry considerations, which provide insights into the electrochemical and thermal stability, charge/discharge mechanisms, structural phase transitions, oxygen evolution, cationic migration and origins of kinetic hindrances.

A legitimate question, however, regards the best way to combine such a chemist’s approach with the spectacular advances in characterization techniques and computational methods so as to be more prolific in identifying practical electrode materials. The design of battery materials has benefited from tremendous progress in computational techniques (first of all, based on density functional theory (DFT) and molecular dynamics (MD)) which greatly contributed to interlinking the “Atoms & Ions” and “Crystal Structure” sectors in Fig. 1, as reflected in selected reviews^2,3^. Great hopes have been placed in the predictive capabilities of the high-throughput materials genome initiative, being nowadays empowered with artificial intelligence (AI)^4^. The materials genome approach relies on computational electronic structure methods^5^ while AI is based on mining, manipulating and reasoning from what is now called Big Data and building artificial neural networks to deduce regularities^6^. So, should our write-up stop here in case of looking old-fashioned? Certainly not. The material-oriented research is still largely driven by simple ideas, hence importance of conceptual models and generalizations. This is what this paper tries to illustrate and discuss as personally perceived.

## Electronic structure and electrochemically induced redox reactions

The redox properties of the insertion materials are best considered in terms of their band structure, which is inherited from the overlapping atomic M *nd* orbitals and L *n*′*p* orbitals, as presented in Box 1, panel a, b. Confining to L = oxygen for clarity, the relative energy of the (M–O)* and (M–O) bands changes concomitantly with the (Δ-*U*/2) term, where Δ and *U* are the charge transfer and *d-d* Coulomb interaction energies, respectively (Box 1, panel d). High Δ >> *U* favors the classical insertion reaction associated with the cationic M^(k+1)+^/M^k+^ redox couple, in which the relative energy of the (M–O)* band with respect to the 1*s* band in metallic Li governs the electrode redox potential (Box 2), whereas lowering Δ increases involvement of the anionic O 2*p*-like states (Box 1, panel e). The most practical benefit from the anionic redox resides in contribution of non-bonding (O 2*p*)_NB_ orbitals delivering extra electrons on top of those given by the cationic M^(k+1)+^/M^k+^ redox center and, hence, extra capacity. The layered oxides Li_4/3−x_Ni^2+^_x_Mn^4+^_2/3−x_Co^3+^_x_O_2_ termed “Li-rich NMCs” demonstrate a high reversible capacity exceeding 250 mA h g^–1^ that cumulates a cationic redox activity (Ni^2+^ → Ni^3+,4+^ and Co^3+^ → Co^4+^) amounting to ~130 mA h g^–1^ and an anionic redox activity of ~120 mA h g^–1^ at potentials above 4.5 V vs. Li^+^/Li. Pushing the Li removal to its maximum provokes a structural instability of the oxidized oxygen accompanied by the release of O_2_, concomitantly with other practical difficulties, such as sluggish kinetics, voltage fade and hysteresis^7^. Curing this problem calls for a fundamental understanding of the Li-driven anionic redox reactions.

**Fig. 2:**
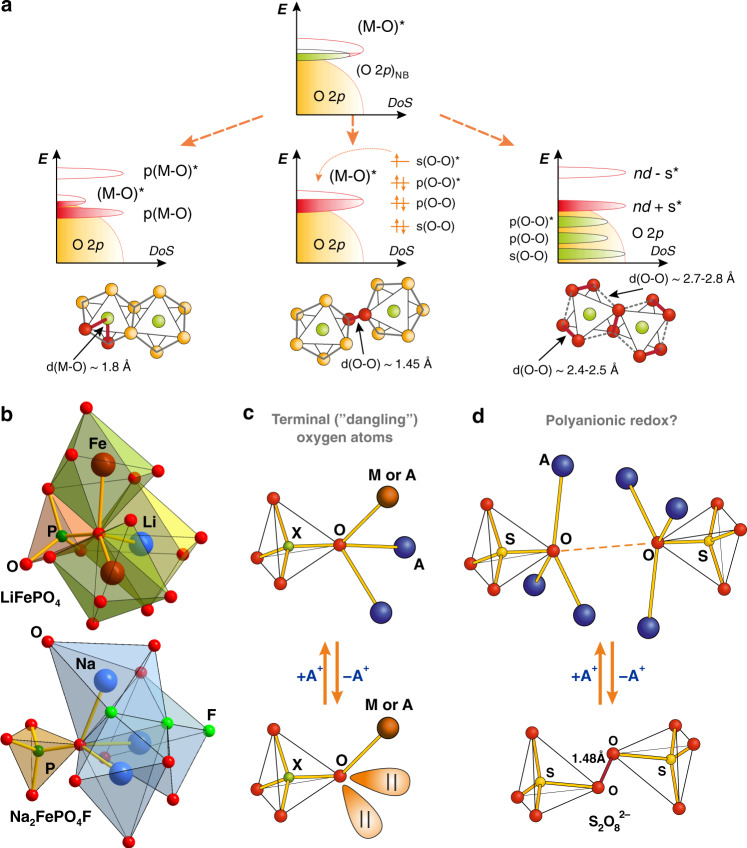
Anionic redox in metal-ion battery cathodes. **a** Stabilization of oxidized oxygen species (from left to right): strengthening of the M–O π-bonds, catenation of oxygens in the (O–O)^2−^ peroxogroups and cooperative distortion of the anionic framework due to a reductive coupling mechanism. sp^3^-Hybridized oxygen orbitals as lone pairs in the polyanion cathode structures: **b** Local coordination environment of selected oxygens in the LiFePO_4_ and Na_2_FePO_4_F structures. **c** Deintercalation of two neighbouring A^+^ alkali cations from the oxo-centered tetrahedron leaves two lone electron pairs residing on the O atom at the center of this tetrahedron. **d** Configuration with two oxo-centered tetrahedra linked to sulfate groups and formation of peroxodisulfate anion S_2_O_8_^2−^ upon deintercalation of the A^+^ cations.

In order to stabilize the highly oxidized oxygen species we need to increase their tightness to the lattice, hence calling for exploration of possible bonding mechanisms. Hong et al. suggested that in Sn-doped Li_2_IrO_3_ the anionic redox leads to π-bonding between Ir 5*d* and O 2*p* orbitals at the cost of shortening the Ir-O interatomic distance to <1.8 Å (Fig.2a, left)^8^. However, if π-bonding might look plausible for the 4*d* and 5*d* transition metals with diffuse *d*-orbitals, it should be less relevant for the 3*d* transition metals as their rather compact *d*-orbitals do not allow for a sufficient π-type overlap with the O 2*p* orbitals. Alternatively, the oxidized oxygens could provoke the anionic catenation mechanism being linked into the O–O dimers similar to that suggested by J. Rouxel for sulfides^9^. Here, pushing the oxidation of the O 2*p* non-bonding states implies formation of covalent O-O bonds and corresponding localized σ, π, π*, σ* molecular orbitals (Fig. 2a, middle). The holes reside in the high-energy σ(O–O)*-antibonding orbital pertaining to the (O_2_)^n−^ dimers with short interatomic distance of ~1.44 Å (similar to that in the true (O–O)^2−^ peroxogroup) bridging two neighboring metal-oxygen octahedra either within the same M–O layer or between the adjacent M–O layers^10,11^. These peroxogroups are associated with the cationic migration from the M positions to the vacant Li positions leaving “undercoordinated” oxygens which use their 2*p* orbitals to bind with each other.

In case of the transition metal sulfides, owing to the lower chemical hardness of S^2−^ with respect to O^2−^, the sulfur catenation into disulfide (S_2_)^2−^ groups is more pronounced as realized in TiS_3_ = Ti^4+^S^2−^(S_2_)^2−^ and FeS_2_ = Fe^2+^(S_2_)^2−^^12,13^. Li insertion is assumed to break the S-S bond resulting in the Li_2_Ti^4+^(S^2−^)_3_ and Li_2_Fe^2+^(S^2−^)_2_ intercalated structures. However, the long-believed anionic redox intercalation chemistry related to forming/breaking polysulfide S-S bonds still needs a solid crystallographic confirmation. In a broader context, such redox chemistry is recently demonstrated for the low-temperature insertion of Cu into La_2_O_2_S_2,_ Ba_2_S_2_F_2,_ LaSe_2_, BaS_2_ containing (L_2_)^2−^ (L = S, Se) dimers to obtain the Cu_2_La_2_O_2_S_2,_ Ba_2_Cu_2_S_2_F_2_, LaCuSe_2_ and BaCu_2_S_2_ phases^14^. Through this insertion, metallic Cu donates electrons from 3*d* orbitals to low-lying σ* orbitals of the (L_2_)^2−^ dimers causing their cleavage. These findings open up a new synthetic strategy worth being explored for designing novel transition metal compounds from precursors containing polyanionic redox centers.

Although the anionic redox scenarios enlisting the M–O π-bonding or the O–O covalent bonding look plausible, they still call for an unequivocal proof of the drastic shortening of the M–O bonds or O–O bonds. Instead, the crystallographic data point to a cooperative deformation of the octahedral framework that was theoretically and experimentally confirmed in both α and β-polymorphs of Li_2_IrO_3_^15,16^. This deformation, as deduced by transmission electron microscopy and Rietveld refinements, consists in a trigonal prismatic distortion of the MO_6_ octahedra that splits the O–O distances into three long and three short (~2.4–2.5 Å) ones (Fig. 2a, right). Such a shortening of the O–O distances triggers mixing between the partially filled σ(O-O)*-antibonding orbitals and the (M–O)* states resulting in the filled *nd*+σ* and empty *nd*-σ* bonding and antibonding states, respectively (Fig. 2a, right)^11,17^ that is referred to as “reductive coupling”. While a corresponding mechanism has been confirmed for the Li-rich layered oxides of the 4*d* and 5*d* transition metals, its occurrence remains questionable for the 3*d* transition metal oxides as theoretically demonstrated for Li_2−x_MnO_3_^18^, but not impossible. Surprisingly, it was found in the recently discovered pyrite-structured FeO_2_, which exists at high pressure (76 GPa) and high temperature (1800 K)^19,20^. This suggests that the feasibility of reductive coupling increases with either a spatial extension of the M *nd* orbitals, or, otherwise, a shortening of the M–O distances by applying external pressure. The effect of pressure can be mimicked with epitaxial strain compression in thin films that is known as a tool to modify physical properties. Thus, finding the right epitaxial relations and lattice mismatch could enable stable and reversible oxygen redox due to the reductive coupling in thin film or even 3D electrodes based on 3*d* transition metal oxides.

The non-bonding oxygen states in the Li-rich layered oxides are associated with the unhybridized (O 2*p*)_NB_ orbitals (also called “lone pairs”) aligned along the linear Li-O-Li bridges^21^, but other types of anionic “lone pairs” as electron donors have also been considered in Li-rich layered oxides. This was successfully done in Na-based layered oxides with Mg^2+^ and Zn^2+^, which form ionic bonds with oxygen and by the same token can form peroxide compounds (MgO_2_, ZnO_2_)^17,22^. In LiFePO_4_, the “stellar member” of the polyanionic positive electrode materials, oxygens sit in the oxo-centered tetrahedra (Fig. 2b). Their *sp*^3^ orbitals mix timidly with the Fe 3*d* states rendering LiFePO_4_ a Mott insulator, but upon delithiation the covalency of the Fe-O bonds increases converting FePO_4_ into a charge transfer insulator with almost pure O 2*p* states at the Fermi level^23,24^. This electron density forms domains of lone electron pairs (Fig. 2c). Even more interesting are the polyanionic structures exemplified with (but not restricted to) fluoride-phosphates A_2_MPO_4_F (A = Li, Na, M = Fe, Co), within which the tetrahedral oxygens are not even bound to the transition metal; the reasons why they are termed “dangling” or “semilabile” (Fig. 2b)^25,26^. The lone pairs of these oxygens are equivalent to oxygen non-bonding states and could potentially act as electron sources.

Although these electrons are believed to be too low in energy, hope must prevail as evidenced by deintercalation of two Li^+^ cations from Li_2_FeSiO_4_ apparently assisted by hole formation at the lone oxygen orbitals^27^. Similar to the anionic redox in oxides, the oxidized oxygen atoms of two neighboring polyanion groups, such as two sulfate groups with the “dangling” oxygens (Fig. 2d), could form a peroxobridge through covalent bonding that is well known as oxidation of sodium hydrosulfate NaHSO_4_ into peroxodisulfate Na_2_S_2_O_8_: 2SO_4_^2−^ → S_2_O_8_^2−^ +2e^−^, *E*^o^ = 2.01 V vs. SHE (estimated as 5.05 V vs. Li^+^/Li). Although the redox potential is beyond the stability window of the standard carbonate-based electrolytes, recent report on highly fluorinated electrolytes stable up to 5.6 V vs. Li/Li^+^ might curb this difficulty^28^. In short, it is not therefore impossible to envisage that the polyanionic redox process could be triggered upon removal of Li, hence justifying further exploration.

One conclusion to be drawn from this brief overview is that, although the general picture of the anionic redox is well established at least with respect to the importance of the O 2*p* non-bonding states, we are still far from a complete understanding. The main reason is rooted in difficulties of getting precise knowledge on the structure at the end of charge and its evolution during charge-discharge, although the fundamental understanding received from such data is worth the efforts^29^. The structural complexity of layered oxides (abundant stacking faults, cations intermixing, gliding close packed layers upon charge/discharge, cation migration between octahedral/tetrahedral interstices) poses serious obstacles for decent crystallographic studies. Moreover, it is intriguing why there is a relatively small number of extended solids having transition metals coordinated by (O–O)^2−^ peroxogroup alike WO_2_(O_2_)H_2_O^30^ while the panel of materials showing anionic redox activity is rapidly growing? An explanation from a crystal structure and chemical bonding standpoint could really benefit the anionic redox field. Additional insights are also necessary for designing chemical strategies that could help in taking high capacity anionic redox cathodes from the labs into market.

Box 1 Molecular orbitals and band theory in application to electrode materials In the majority of the insertion compounds for metal-ion batteries a transition metal cation M^k+^ is octahedrally coordinated with ligands L that results in a schematic band structure deduced from the overlapping atomic M orbitals with L *n’p* orbitals (panel a), whereas part of the L *n*′*p* orbitals are not capable of bonding overlap by symmetry and remain non-bonding (NB). The “orbitals of interest”, i.e. those determining the redox properties, comprise the bonding orbitals with a major L contribution (yellow, panel **a**) and anti-bonding *e*_g_ and *t*_2g_ orbitals with a major M *nd* impact (pink, panel **a**). Being placed into a periodic crystal potential, these groups of molecular orbitals give rise to the electronic “bands of interest”, which are the (M–L)* antibonding and (M–L) bonding bands, and the non-bonding (L *n’p*)_NB_ band (panel **b**). However, in case of strong electron-electron correlations one needs to consider the band structure in terms of the Zaanen-Sawatzky-Allen theory^75^. The *d-d* Coulomb repulsion splits the partially occupied (M–O)* band into empty upper and filled lower Hubbard bands (UHB and LHB, respectively) with an energy separation *U* that is the energy penalty for *d*^n^*d*^n^ → *d*^n+1^*d*^n−1^ electron transfer between adjacent M sites giving rise to a Mott-Hubbard insulator with the bandgap inside the (M–L)* states (panel **c**). In case of the large *U*, the highest-energy filled band is L *n*′*p*, and virtual electron transfer occurs between ligand and metal, defined by a charge-transfer (CT) energy Δ, which is the energy cost of moving an electron from ligand and placing it at the M *nd* orbitals *d*^n^→*d*^n+1^*L* (*L* is the ligand hole). In the Mott-Hubbard regime *U* < Δ, but if Δ < *U*, a CT insulator is realized with the bandgap between the UHB and L *n’p* bands. Δ and *U* can roughly be traced with the ionic model (panel **d**)^96^. Solid-state chemists manipulate these terms by tuning the electron affinity of the M cations that increases with either their electronegativity or with formal oxidation state. Neglecting electron-electron correlations for the sake of simplification, upon Li^+^ removal and in absence of drastic structural transitions, decreasing the Madelung term results in a decrease and increase in energy of the cationic (M–L)* and anionic L *n*′*p* bands, respectively, hence lowering Δ_,_ but the holes still reside in the cationic (M–L)* band (panel **e**, points 1–3). Thus, the redox reaction is mostly cationic with the M^k+^ cation acting as the major redox center. For the M^k+^ cations with higher electronegativity at the end of the M^(k+1)+^/M^k+^ cationic capacity, the L *n’p* orbitals are pushed towards the Fermi level equalizing the contributions of the M *nd* and L *n*′*p* states (panel **e**, point 4). The holes equally belong to the cationic and anionic sublattices. The increased hybridization of the M *nd* and L *n’p* states implies high covalency of the M–L bonding rendering the L ligand a participant of the charge transfer process where holes partially reside at the L *n’p* states that, however, does not increase the overall number of electrons donated by the cathode, as the overall number of holes remains the same. Partial oxygen oxidation in contrast can lead to capacity advantages, as realized for the rock-salt based complex transition metal compounds, which by specific structural an stoichiometry reasons (L/M ratio >1) involve redox-active non-bonding (L *n*′*p*)_NB_ orbitals^21^. Accordingly, upon Li^+^ removal, extra capacity is gained from the localized (L *n*′*p*)_NB_ anionic states in addition to that coming from the (M–L)* band (panel **e**, point 5).

Box 2 Crystal chemistry rationale behind the operating voltage of metal-ion battery electrodes In the general case of M–L (L = ligand) bonding, the electrochemical redox potential is determined by the position of the (M–L)* band that hosts the Fermi level relative to the Li 1*s* band. The energy difference between (M–L)* and (M–L) can be expressed as a function of the overlap integral, *S*^2^, and electronegativity difference, Δχ, between M and L (panel **a**). The higher Δχ, the more ionic the M–L bond. This lowers the *S*^2^/Δχ and consequently the (M–L)* band such that the redox potential increases.

Introducing a complex polyanion group XL_n_^m−^ (X = Si, P, S, B, C etc.) offers another degree of freedom in tuning the electrode potential of electrode material by varying ionocovalency of the M–L bonds by means of “inductive effect” (panel **a**, right). In terms of the band structure, placing a highly electronegative X cation lowers the energy of (M–L)* antibonding states and Fermi level thus enlarging the energy gap between Fermi level in the cathode and the Li^+^/Li redox couple (panel **b**). Further on, the inductive effect is not simply governed by the chemical nature of X: a more accurate prediction of the electrode potential can be made based on the understanding of how the entire coordination environment of the transition metal–ion affects the covalency/ionicity of the M–L bonds including the distances with the nearest neighbors and the contribution of cation–cation electrostatic repulsion^97^. The increased electrostatic repulsion through shorter cation–cation interactions typically results in enlarging of the M–L bond distances and consequently increases their ionicity leading to a higher potential of the M^(k+1)+^/M^k+^ redox couple.

At the same time changing the coordination number of the M cation (from ML_4_ tetrahedron through ML_5_ trigonal bipyramid to ML_6_ octahedron) can render the M–L bond more ionic thus raising the M^(k+1)+^/M^k+^ redox potential. The number of A^+^ alkali cations nesting in a close proximity to the redox center also affects the M^(k+1)+^/M^k+^ redox potential that is considered as a “secondary” inductive effect (panel **c**). The A–L bonding increases the ionic character of the M–L bonds through a similar mechanism as to the XL_n_^m−^ polyanions. In practice, shifting the ionocovalency of the M–L bond through the inductive effect appears a useful tool to tune the redox potentials in quite a broad range. For instance, a cumulative inductive effect of fluoride and phosphate anion species in KTiPO_4_F allowed for a substantial boost of the Ti^4+^/Ti^3+^ redox potential up to 3.6 V vs. K^+^/K turning it into a prospective cathode despite the well-establish perception of Ti-containing compounds as “only-anode” materials^98^.

## Ionic transport and defects

So far, considering a battery electrode as an electron reservoir was instructive for establishing a solid connection between the chemical bonding, electronic structure and charge-discharge mechanisms. However, the mobility of the shuttle cations is another important asset for practical implementation of the electrode materials as it largely determines the battery’s power. Besides electrode materials, inorganic ionic conductors are becoming of paramount importance for the development of solid-state batteries to meet electric vehicle user’s demands for safer and greater autonomy batteries. One of the bottlenecks towards achieving this goal is enhancing ionic conductivity and chemical/electrochemical stability against other battery components^31^. Understanding defect chemistry turns out to be as important as the crystalline matrix to design efficient ionic conductors.

Both battery electrodes and Li(Na)-based inorganic electrolytes can be described as an anionic framework in which mobile cations hop between sites through interconnected diffusion channels. Their ionic conductivity (σ), which is a thermally activated process following the Arrhenius law, is governed by the charge (*q*), the number (*n*) of mobile carriers (ions, interstitials, vacancies) and the activation hopping energy (*E*_a_) for the conducting ion. The latter is the sum of the energies to create the defect and to overcome the energy barrier for the ion migration that depends on the local bonding environment and interconnectivity between available sites in the anionic sublattice. Within a simple ionic model, the electrostatic interactions of neighboring ions together with the free structural volume for ion migration can be estimated fairly well beyond time-consuming DFT-based methods. These approaches, briefly summarized in Box 3, enlist bond valence maps or energy landscapes and crystal space analysis with Voronoi–Dirichlet partitioning^32^. The latter provides the network of diffusion channels and large local spaces promoting fast diffusion. The former delivers a tentative estimate of the energy barriers for ion diffusion, ranking materials by the energetics of this space. The polarizability of the anion sublattice can also be accounted for by appropriate modification of the bond valence parameters^33^. These approaches are typically applied for pre-screening of solid electrolytes and electrode materials being supplemented with simulations of higher accuracy for precise evaluation of the most promising selected examples. As a result, many previously untested compounds with anticipated ion-diffusion properties have been identified as prospective candidates for further investigation not only for the Li-ion conductors, but also for Na-, K-based ones^34–36^.

**Fig. 3:**
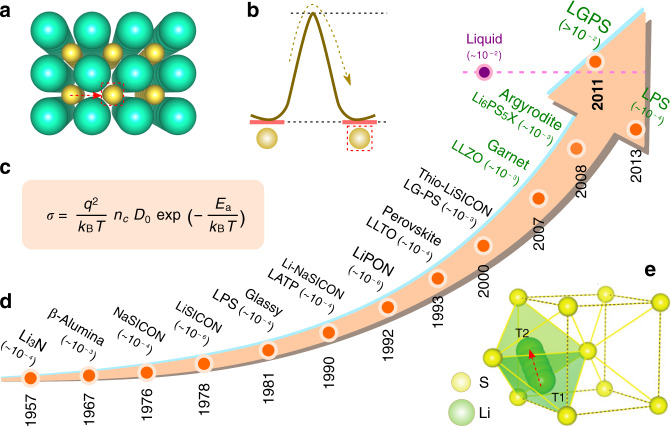
Quest for highest Li-ion conductivity in inorganic solids. The overall equation defining the ionic conductivity σ is shown together with a schematic of the activation energy barrier corresponding to the hopping of ions from site to site within the crystal structure (**a**, **b**) together with the most favorable migration path between two tetrahedral sites T_1_ and T_2_ in the *b.c.c.* anionic structure (**c**). Development of solid electrolytes over times together with their superionic conductivities (in S·cm^−1^) indicated in the parentheses (**d**). The legend: NaSICON: Na_3_Zr_2_PSi_2_O_12_; LiSICON: Li_14_ZnGe_4_O_16_; Glassy LPS: Li_2_S–P_2_S_5_; LATP: Li_1.3_Al_0.3_Ti_1.7_(PO_4_)_3_; LiPON: Li_2.98_PO_3.30_N_0.46_; LLTO: Li_x_La_2/3−x/3_TiO_3_; LG-PS: Li_4–x_Ge_1–x_P_x_S_4_; LLZO: Li_7_La_3_Zr_2_O_12_; LGPS: Li_10_GeP_2_S_12_; LPS: Li_3_PS_4_.

Trying to identify rules to relate the structure to the ionic conductivity, it has long been recognized that the best ionic conductors (α-AgI, Li_10_GeP_2_S_12_, Li_7_P_3_S_11_, etc.) have highly polarizable anionic frameworks adopting a body-centered cubic packing (Fig. 3). Such specificity is not fortuitous as recently rationalized by combining a structural matching algorithm with DFT calculations demonstrating that the *b.c.c*. framework allows the Li^+^ ions to migrate through the interconnected tetrahedral sites with a lower activation barrier compared to *h.c.p*. or *f.c.c*. frameworks^37^. The same trend holds for other monovalent (Na^+^) or multivalent (Mg^2+^) ions. However, since *b.c.c*. anion frameworks are less common than the *h.c.p*. or *f.c.c*. ones, high ionic conductivity should be limited to a small number of compounds. Gladly, there are a few exceptions to the rules with namely the argyrodite-type Li_7_PS_6_ and its halide-substituted derivatives, which show high ionic conductivity while not having a *b.c.c*. anionic framework but still containing tetrahedral sites for the mobile ions. Such insights open perspective for further exploration among both sulfides and oxides.

In the quest for a higher ionic conductivity, lattice stiffness of a few conductors has been monitored by probing the optical and acoustic phonon modes, as well as the phonon density of states via inelastic neutron scattering. Correlating these metrics with the experimental ionic transport reveals that lattice dynamics serves as a useful descriptor for better ionic conductors^38^. Among others, this effect is conveyed in Zn-substituted Na_3_PO_4_ ionic conductors that comprise dynamic rotational disorders in the anionic sublattice enhancing ionic conductivity by the “paddle-wheel” mechanism^39^, where the cation hopping is accompanied by a simultaneous rotation of the anion lowering the activation barrier^40^. A recent study on Na^+^-ion migration and PS_4_^3−^ rotation in Na_11_Sn_2_PS_12_ revealed their intimate coupling and further confirmed the “paddle-wheel” mechanism as a transient opening of a migration window along the Na^+^ transport channels^41^. The “paddle-wheel” mechanism can be intrinsic not only in the crystalline solids with the rotational disorder of the polyanion groups, but also in amorphous solid electrolytes^42^.

Whatever the underlying structure is, it must be recognized that ionic conductivity can also be enhanced with a configurational or occupational disorder resulting from either vacancies or interstitials. Temperature is another elegant way to introduce the configurational disorder, hence explaining why in numerous solid electrolytes the high-temperature (HT) and highly disordered polymorphs always show best ionic conductivity. An illustrative example is the garnet-type Li_7_La_3_Zr_2_O_12_ which is tetragonal and a poor ionic conductor at room temperature (RT) (*σ*_RT_ ~ 10^−6^ S cm^−1^) while the HT cubic phase is a good ionic conductor with an extrapolated *σ*_RT_ of ~10^−4^ S cm^−1^ due to the high Li disorder^43^. Quenching together with chemical substitution are the common strategies to stabilize the HT disordered polymorphs at RT. The highest Li ionic conductivity of *σ*_RT_ ~ 1.4 × 10^−3^ S cm^−1^ among oxides was achieved for the inherently disordered Ga-substituted cubic Li_6.25_Ga_0.25_La_3_Zr_2_O_12_ garnet^44^ providing the absence of grain boundaries which most frequently negatively affect ionic conductivity. Such boundaries usually originate from crystallites of different orientation in polycrystalline samples and more frequently from minute amounts of surficial phase having either structure, composition or both, different from the grain material.

More spacious and polarizable framework is preferable for ionic conduction, as it weakens the Li-anion bonds reducing *E*_a_. Along that line, the replacement of O^2−^ with larger and more polarizable S^2−^ widens the diffusion pathways and improves the conductivity by several orders of magnitude. The research on sulfide-based ionic conductors has recently been accelerated owing to the pioneering work of Kanno *et al* suggesting highly conducting (1-x)Li_4_GeS_4_–xLi_3_PS_4_ phases guided by a tendency of increasing ionic conductivity with the number of mobile charge carriers, namely Li vacancies in the Li_4−x_Ge_1−x_P_x_S_4_ solid solutions due to the heterovalent P^5+^ for Ge^4+^ substitution^45^. The actual crystal structure of the Li_10_GeP_2_S_12_ (or Li_3.33_Ge_0.33_P_0.67_S_4_) phase with the highest ionic conductivity *σ*_RT_ ~ 10^−2^ S cm^−1^ is, however, different from the parent Li_4_GeS_4_ and Li_3_PS_4_ structures^46^. Further optimization via dual cationic (Si for P) and anionic (Cl for S) substitutions resulted in Li_9.54_Si_1.74_P_1.44_S_11.7_Cl_0.3_ with *σ*_RT_ ~ 2 × 10^−2^ S cm^−1^ comparable to that in the Li-based liquid electrolytes^47^. This combination of ligands also provides high ionic conductivities in the argyrodite-type phases Li_6_PS_5_X (X = Cl, Br, I) with the maximum *σ*_RT_ ~ 0.94 × 10^−2^ S cm^−1^ for Li_5.5_PS_4.5_Cl_1.5_^48^. Equally, the high temperature β-Li_3_PS_4_ polymorph with *σ*_RT_ ~ 10^−3^ S cm^−1^ can be synthesized at low temperature (<150 °C) by nano-structuring via wet-chemical synthesis^49^. However, inherent drawbacks of sulfide-based electrolytes are nested in their limited electrochemical stability which necessitates the use of coatings when placed in contact with oxide positive electrodes, but also in their moisture reactivity which complicates their handling. To alleviate this issue great efforts are placed towards the design of oxysulfides that is a real challenge for solid-state chemists. A viable alternative is provided through revisiting ion transport uncovered back to the 1930’s in LiX (X = F, Cl, Br, I) that has led to Li-based ternary halides Li_3_YX_6_ (X = Cl, Br) with ionic conductivities of 0.07–1.7 × 10^−3^ S cm^−1^ after ball milling. Moreover, in terms of practical benefit these ternary halides are stable at high potential as demonstrated with an electrochemically workable (Li_3_YCl_6_/LiCoO_2_) cell without pre-coating of the cathode^50^.

At this stage, it is worth recalling that the site energies and migration barriers in the metal-ion battery electrodes may vary upon continuously changing guest cation concentration. Herein, the concentration-dependence of the diffusion activation barriers stems from the size variation of the interstitial voids along the A-cation hopping trajectory due to changing the electronic state of the M cations, their ionic radii and electronically-driven polyhedra distortions. Layered oxide cathodes show many of these aspects that is often accompanied by a short-range migration of the transition metal cations to octahedral positions in the Li layer or to tetrahedral interstices coupled with the anionic redox^29,51^. This migration increases the Li diffusion barriers causing a contraction of the Li layer thickness^52^. Being only partially reversible, the migration gradually transforms the layered structure to spinel-like or disordered rock-salt like structures raising the cell impedance^53^. However, even with a high degree of the Li–M disorder, Li-rich rock-salt oxides can still demonstrate measurable Li diffusion if the Li concentration enables the percolated diffusion paths^54^. The dynamic antisite disorder can equally be induced electrochemically in polyanionic cathode materials possessing the polyanionic groups with “semilabile” oxygen atoms. By extraction of the A^+^ cations these oxygens experience severe underbonding due to the depleted coordination environment (Fig. 2b) that can be partially compensated by migration of the M cation to the vacant A positions^26^. At the same time, the undercoordinated semilabile oxygens with their *sp*^3^ lone electron pairs carry excessive negative charge acting as traps for the A^+^ cations and increasing their hopping barriers^25,55^.

Lastly, the role of the extended defects in ionic transport in solid electrolytes, as mentioned before, is well recognized (particularly the role of grain boundaries), and also acknowledged in the electrode materials. The Li^+^ transport in LiCoO_2_ is demonstrated to be sensitive to diffusivity along the grain boundaries, grain size and spatial distribution of the grains^56^. For instance, a three orders larger diffusion coefficient is expected for Li^+^ ions traveling along the coherent Σ2 grain boundary in LiCoO_2_ compared to that in the direction across the boundary^57^. These examples call for a better understanding of the grain boundary structure and developing advanced chemical approaches to control the grains and grain boundaries in electroactive materials and solid electrolytes.

Box 3. Topology and bond valence approaches to cation diffusion Solid-state chemistry methods based on crystal structure analysis can be applied for both electrode and solid electrolyte materials to probe potential ion migration pathways, find intercalation sites, and roughly estimate the activation energy of ion migration within reasonably short time. Among them are purely geometry-topological approaches such as Voronoi–Dirichlet polyhedra (VDP) partition, which deals with crystallographical space, and electrostatics-based methods known as “bond-valence” (BV)^32^.

According to VDP, the crystal space can be split into atoms and voids subspaces. VDPs are built only around so-called “framework” atoms, which remain immobile during ion migration. The vertices and edges of VDP (panel **a**, top) indicate elementary voids and channels sorted by their radii (Rsd, and Rad, respectively). Only significant voids and channels with sufficiently large Rsd and Rad are suitable for mobile ions. Such downselection results in a continuous system represented by curved 1D pathways for LiFePO_4_.

In predominantly ionic and covalent compounds, the sum of bond valences (BVS) of a specific ion should be equal to its valence (formal ionic charge), and can be calculated according the formula (1), panel **a**, where *d*_*j*_ is the bond length, *R*_*0*_ and *b* are tabulated constants. The BVS concept is widely recognized as a tool for locating light elements in the structure and identifying mobile ion migration pathways. For this purpose, a BVS value is calculated for each point of a unit cell partitioned into a 3D grid with a given step (<0.2 Å) resulting in a BVS map (panel **a**, middle). The key parameter—BVS mismatch (BVSM)—limits the deviation of all BVS values from the reference (i.e. 1 for Li and other alkali ions). The higher BVSM in a particular area, the lower possibility for a mobile ion to pass through it^99^. Once the sites with low BVSM percolate, ionic conduction is likely to be possible. An important advantage of BVSM is not only low time cost (panel **b**), but also easier treatment of non-stoichiometric compounds (sites with multiple elements or partial occupancies, disorder) by adding corresponding normalizing coefficients to the formula (1). The BVSM values can be translated into energy-related values using a Morse-type interaction potential. Such an approach is called bond valence energy landscape (BVEL)^100^, which is sometimes referred to as bond-valence site energy. With the BVSM input, the site energy, *E(A)*, can be estimated according to the formula (2), panel **a**^100^, where *E*_*asym*_—the energy penalty due to the coordination asymmetry, and *E*_rep_—the electrostatic (Coulomb) repulsion, *V*_id_—expected valence of the A atom, *D*_*0*_ and *g*—empirical coefficients. The BVEL approach might reveal energetically favorable sites for ions or define migration pathways characterized by an activation energy *E*_act_, which can be treated as a migration barrier of a mobile ion. Since BVSM and BVEL deal with a static crystal structure and the relaxation of the surrounding atoms during migration is disregarded, the BVEL migration barrier is generally higher than the experimental one. Nevertheless, substantial literature data for monovalent ions validate a steady correlation between experimental, computational (DFT and MD), and BVEL activation energies. Noteworthy, the solid-state methods also provide a significant gain in time and cost (panel **b**).

## Advances in diffraction, imaging and spectroscopic techniques

**Fig. 4:**
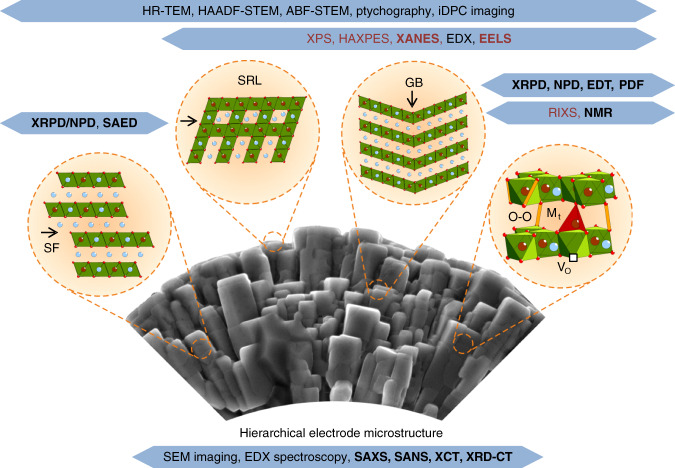
Digging into the crystal structure and microstructure of battery electrodes. Progress in solid state electrochemistry have greatly benefit from establishing robust structural-electronic-electrochemical relationships via the prolific improvement of available diffraction, imaging and spectroscopic characterization techniques and enabling many of them to act in an operando/in situ mode. They target various local structural aspects (vacancies, defects, stacking faults, structure distortions) as well as chemical and electronic aspects. This figure should serve as a guide to the reader for selecting the most suitable type of measurements dealing with a specific problem regarding crystal structure, chemical bonding, electronic structure and composition (as exemplified with the layered rock-salt type oxides). The legend: SF stacking faults; SRL surface reconstruction layer; GB grain boundaries; HR-TEM: high resolution transmission electron microscopy; HAADF-STEM high angle annular dark field scanning transmission electron microscopy; ABF-STEM annular bright field scanning transmission electron microscopy; iDPC integrated differential phase contrast; XPS X-ray photoelectron spectroscopy; HAXPES hard X-ray photoelectron spectroscopy; XANES X-ray absorption near edge structure; EELS electron energy loss spectroscopy; XRPD X-ray powder diffraction; NPD neutron powder diffraction; EDT electron diffraction tomography; SAED selected area electron diffraction; PDF pair distribution function analysis; RIXS resonant inelastic X-ray scattering; NMR: nuclear magnetic resonance spectroscopy; SAXS, SANS: small angle X-ray/neutron scattering; XCT X-ray computed tomography; XRD-CT X-ray diffraction computed tomography. The techniques to probe atomic arrangement are shown in black, those targeting the electronic states and bonding are in red. The techniques in bold can be applied in situ or operando.

In spite of a tremendous progress in understanding the metal-ion electrochemical systems conveyed in the previous sections, the extreme complexity of electrodes and solid electrolytes still does not allow retrieving full details on the bulk and defect structures and their evolution upon battery life cycle which too often leads to hypotheses on the exact nature of their electrochemical behavior based on incomplete data. Chemists have at hand powerful diffraction and spectroscopic techniques to interrogate the materials locally and in the bulk (Fig. 4), but we should not miss the advantages offered by the ongoing development of new methods addressing the specific challenges in the metal-ion batteries. A comprehensive overview of the characterization techniques dedicated to fast ionic conductors is available in a recent review^58^, thus we keep focus at the intercalation materials below.

**Fig. 5:**
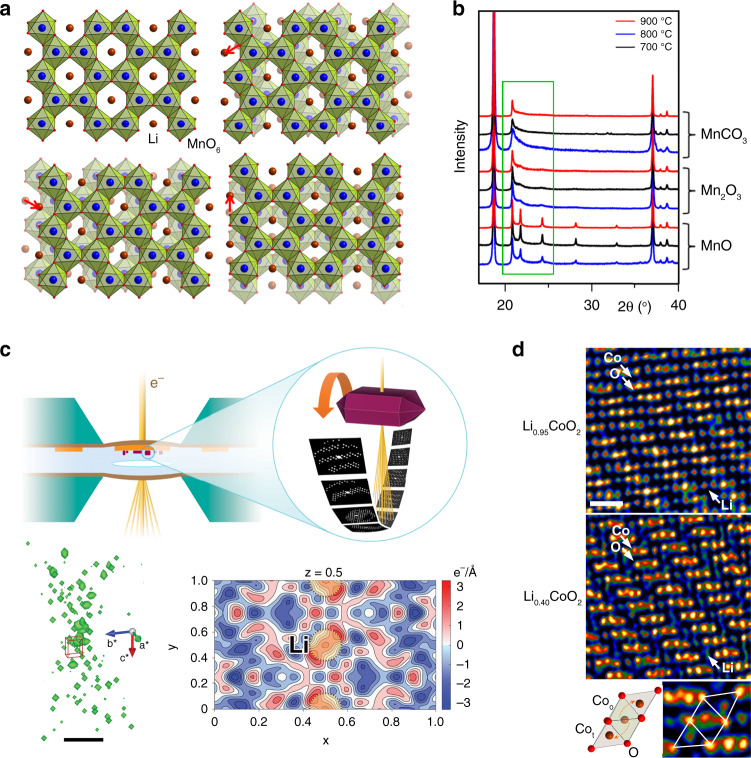
Selected advances in crystallographic characterization of the electrode materials. **a** The structure of “honeycomb”-ordered layers in the Li-rich layered oxides (exemplified with Li_2_MnO_3_) and three energetically equivalent lateral displacements of the adjacent layers giving rise to the stacking disorder. **b** Profiles of the reflections in the XRPD pattern of Li_2_MnO_3_ prepared at different temperatures and from different precursors. The profiles of the reflections originating from the “honeycomb” ordering (outlined in green) are affected by stacking faults in various concentrations (reproduced from ref. ^61^ with permission from the Royal Society of Chemistry). **c** Electron diffraction tomography experiment in a TEM cell with liquid electrolyte and Si_3_N_4_ windows: scheme of the cell and data collection procedure, 3D reciprocal space reconstruction (domains of diffracted intensity at the Bragg positions are shown in green) and difference Fourier map showing the Li positions in the LiFePO_4_ structure. Scale bar is 5 nm^−1^ (reproduced from ref. ^66^ with permission from the American Chemical Society). **d** Projected charge density dDPC maps of Li_0.95_CoO_2_ and Li_0.4_CoO_2_: note clear reduction of the charge density at the Li layers. Defects due to migration of the Co atoms from its native octahedral sites (Co_O_) to the tetrahedral interstices (Co_t_) are clearly seen in the enlarged part of the image of the charged Li_0.4_CoO_2_ material. Scale bar is 0.5 nm.

X-ray and neutron powder diffraction (XRPD and NPD) that can be done in either ex situ or in situ modes using dedicated electrochemical cells, enables to determine precisely the crystal structures of electrode materials. An important obstacle pertaining to such techniques is severe distortion of the diffraction intensities and profile shapes by planar defects, such as stacking faults and twins. Li-rich layered oxides Li_1+x_M_1−x_O_2_ compile such a complexity by reuniting within the same structure faulted stacking associated with random lateral displacements of the “honeycomb” Li/M ordering (Fig. 5a, b), faulted close-packing sequence due to the “cubic-to-hexagonal” transformation of the close-packed layers, and incomplete Li/M ordering depending on the x value. Although the theory of diffraction from faulted structures is well-known and corresponding software is developed^59^, it is still rarely used, most probably because of the complexity of modeling faulted stacking sequences, but more so owing to their dynamical behavior upon Li^+^ uptake and removal^60,61^. Tracing the defect structure evolution throughout charge/discharge is still a challenge. Most important, it impedes retrieving precise information on dependence of interatomic distances on the state-of-charge (SoC) limiting our ability to judge on evolution of the chemical bonding. Being sensitive to average long-range order, XRPD and NPD do not probe short-range correlations, and this gap can be closed with total scattering with a pair distribution function (PDF) analysis, which delivers local information on interatomic distances, coordination numbers and disorder, without presuming periodicity^62^. Neutron PDF provides a unique capability to reveal 2D ordering of transition metal cations in the layered oxides and 3D clustering in disordered rock-salt oxides that would be difficult to resolve with other methods because of the closeness of atomic numbers of the 3*d* transition metals^63^. The local structure can be completed with ^6,7^Li or ^23^Na nuclear magnetic resonance (NMR) spectroscopy which demonstrates high sensitivity towards surrounding of alkali cations by transition metals because of the strong dependence of the hyperfine shift on the number and types of the A-O-M interactions with the paramagnetic centers^64^.

Compared to powder diffraction, electron diffraction tomography (EDT) demonstrates a number of advantages being applied to the Li-ion battery electrode materials^65^. Electron diffraction intensities can be collected ex situ from submicron-sized single crystals of the electrode at different SoCs free of conductive additives and binder interference, and used for the crystal structure solution. The relative sensitivity of EDT to lithium compared to that of X-rays scales as ~[Z_heavy_/3]^1/2^, where Z_heavy_ is the atomic number of the heaviest element in the structure, hence enabling a ~3 times better Li detection in the structures like LiCoO_2_ and its derivatives. Thus, Li positions, occupancy factors and antisite disorder can be determined rather precisely^26^. Moreover, EDT is so far the only single-crystal diffraction method to retrieve the crystal structure quantitatively from in situ experiments inside the electron microscope using an electrochemical cell with liquid electrolyte (Fig. 5c)^66^. The advantage of EDT relies in the use of a weak electron beam thus reducing electron dose and minimizing electrolyte decomposition. Further advances are rooted in the full dynamical structure refinement that significantly improves the quality of crystallographic data reaching ~0.02 Å precision in atomic positions^67^ and in treatment of diffuse electron scattering that provides information on planar defects and disorder^68^.

A deeper insight into the local structure requires advanced transmission electron microscopy (TEM) imaging. Aberration-corrected high angle annular dark field scanning TEM (HAADF-STEM) and annular bright field STEM (ABF-STEM) have extensively been used to visualize the M cation migration upon the battery cathode cycling^69^, surface layer reconstruction in layered oxides^51^, antisite defects in LiFePO_4_^70^, O–O dimers formed at anionic redox^15,16^, staged Li-vacancy ordering^71^, and many other local details. However, these techniques require relatively high electron dose to provide images with a reasonable signal-to-noise ratio because the annular detectors used in these methods collect only a small fraction of the electrons interacting with the specimen. Hence, there are risks of creating misleading artefacts for charged cathodes, which are frequently highly instable. Nowadays, a broader availability of fast pixelated or segmented STEM detectors introduced emerging alternative low-dose STEM imaging techniques, such as ptychography and differential phase contrast (DPC). In ptychography, a convergent beam electron diffraction (CBED) pattern is recorded for every scanned point of the specimen with a pixelated electron detector and used for reconstructing an atomic-resolution phase image, which is capable of showing reliably the positions of Li, O and heavier elements even at subpicoampere electron currents^72^. In contrast, during the integrated differential phase contrast (iDPC-STEM) imaging, the “center of mass” shift of intensity in the CBED pattern at every scanned point is recorded with a 4-quadrant STEM detector. iDPC-STEM retrieves direct phase image of the transmitted electron wave providing a linear dependence of the contrast on the atomic number Z that is more advantageous for visualizing light elements than the strong ~Z^2^ dependence in the HAADF-STEM imaging^73^. The DPC images can be recorded at the electron dose at least few orders of magnitude lower than a conventional ABF-STEM image still maintaining a good signal-to-noise ratio. As a proof of concept, Fig. 5d shows experimental differential dDPC charge density maps of the LiCoO_2_ electrode (5% and 60% delithiated). Spectacularly, Li vacancies are clearly visible already at their low concentration, whereas at large delithiation the Li-vacancy ordering is unambiguously detected, and a partial migration of Co to the tetrahedral interstices becomes apparent.

The low-noise imaging at the low electron dose stands as a serious asset for extracting atomic positions with sub-Å precision through a quantitative image processing with the statistical parameter estimation theory since the highest attainable precision depends on both spatial resolution of the microscope and signal-to-noise ratio of the image. Based on fitting intensity at the atomic columns with Gaussian peaks and retrieving the atomic column positions, this approach has already been applied to a number of materials^74^, and availability of the low-dose STEM imaging techniques makes it a perfect tool to study quantitatively the atomic structures of oxidized oxygen species formed due to the anionic redox, clusters of point defects, grain boundaries and interfaces.

Aside from representing a transformational change in design of high capacity electrode materials, the anionic redox has also modified the characterization landscape calling for new approaches to visualize, besides cations, oxygen atoms and more so to underpin the charge compensation mechanism involving superoxo/peroxo-like (O_2_)^*n*−^ species. Hence, a resurge of interest for spectroscopy techniques, from the time of the high-T_c_ superconductors, to probe ligand’s electronic state as described by the Zaanen-Sawatzky-Allen diagram^75^. Among them, lab X-ray photoelectron spectroscopy (XPS) has been the most popular with its main drawback of probing solely the material surface (<8 nm). However, XPS provided the first hint of anionic activity by the presence of an extra O signal located at 530.5 eV in the O 1*s* spectrum of Li-rich layered oxides. Its hastily assignment to the (O_2_)^*n*−^  species became rapidly controversial because its energy position was structure- and charge-independent^76^. A similar issue occurs with the sulfide compounds showing an anionic redox activity. The same uncertainty remains in hard X-ray photoelectron spectroscopy (HAXPES) which by virtue of a higher energy X-ray beam of ~6.9 keV can increase the inelastic mean free path and therefore the bulk sensitivity up to ~30 nm^77^. To tackle this issue soft X-ray absorption spectroscopy (soft-XAS) which probes the unoccupied states at the atomic level was brought into the scene for looking at the O K-edge. While such a technique demonstrated great merits for cations, a serious pitfall with the O K-edge XAS interpretation is encountered when correlating the intensity changes in the pre-edge of the spectra with the holes on oxygen sites, because of hybridization between the O 2*p* and M levels, hence leading to highly controversial results^78^. Beyond the conventional XAS, resonant inelastic X-ray scattering (RIXS), which probes the excited states promoted by the electric dipole transition, is described as the “Holy Grail” to track the evolution of the M and O redox in Li-rich electrodes upon delithiation. Such a confidence is rooted in the appearance of a specific feature at 523.7 eV emission energy which resonates at 531 eV incident energy^79^. As for HAXPES results, this feature was almost material-neutral and assigned to the anionic redox. However, no theoretical evidences have allowed to undoubtedly assigning such a feature to the occurrence of exotic O states. Obviously more rigorous implementations of RIXS are being awaited along with an in-depth theoretical support to further elaborate on the nature of the oxidized oxygen species formed during charge and hopefully to bring a corrected trend/picture to this topic.

## Discussion

Modern solid state chemistry, relying on scientific rules, advanced characterization techniques and being empowered with computational tools, is capable of uniting composition, structure and material’s properties and delivering predictions by following a deductive reasoning. DFT- and MD-based computational methods provide a valuable insight into different aspects of the electrochemical behavior of battery-related materials. However, they are very demanding for computer power and often offer too simplified treatment of the real chemical systems, such as disordered or partially ordered materials, materials with intersite mixing, high concentrations of defects, strong electron correlations etc. Nevertheless, the predictive power of these methods in battery research have resulted in several experimentally confirmed polyanion electrode materials, such as the A_3_MPO_4_CO_3_ (A = Li, Na, M = Fe, Mn) carbonophosphates^80^, LiMoP_2_O_7_ pyrophosphates^81,82^, Li_9_V_3_(P_2_O_7_)_3_(PO_4_)_2_ phosphate-pyrophosphate^83^ and Li_3_Cr_2_(PO_4_)_3_^84,85^. However, considering the complexity of the battery materials, a combination of the solid state chemistry approaches with the computational tools still remains serious assets. Among many other examples, this can be illustrated by recent discoveries of metal-ion battery cathodes triggered by the relatively simple concept of the anionic redox and its crystal chemistry expansions: the Li_2_(M,M′)O_3_ (M = Ru, Ir, M′ = Ti, Sn)^15,16,69^ and Li_3_IrO_4_^86^ oxides with the layered and framework structures, the Li-rich disordered xLi_3_NbO_4_ – (1-x)LiMO_2_ (or Li_2_MO_3_) (M = Mn, Fe, Co, Ni)^87^ and short-range ordered Li_1.2_Mn_0.4_M_0.4_O_2_ (M = Ti, Zr)^63^ rock-salt oxides and Li_1.9_Mn_0.95_O_2.05_F_0.95_ oxyfluoride^88^, the Na-based Mg/Zn-doped layered oxides^17,22^ and Na-based Li-rich layered oxides^89^, Li_1.68_Mn_0.6_O_4−x_F_x_ partially ordered spinels^90^, and, finally, the Li-rich layered Li_1.33–2y/3_Ti_0.67–y/3_Fe_y_S_2_ sulfides^91^. However, the next step towards the materials with practical importance is clearly of high demand.

Most likely, methods based on Big Data would come into play in the near future, where the semiquantitative links between the chemical composition, crystal structure and electrochemical properties might form a playground for computer-driven analysis of such relationships. Nowadays we evidence an explosive growth of applications of the machine-learning methods in solid-state materials science aimed at predictions of material’s structure and properties^92^. The most significant problem foreseen on this way is the limited size of available well-labeled datasets required for building good models and training the neural networks (that could be mitigated by relying on computational datasets and unsupervised learning algorithms^6^). One could argue that there is a massive amount of data already stored in scientific publications, which are exponentially growing within the field of batteries, but the reported metrics are full of incoherency with respect to composition characterization, preparation techniques, specimen preparation, measurement methods causing the data variance and making a large part of reports obsolete. More importantly, the whole battery research community must be structured to provide standardized and reliable data calling for more “quantitative” experimental characterization techniques, particularly with respect to retrieving information on disorder and defects. Whatever, the predicted materials will have to be synthesized after all, which brings us back to the skills and competences in solid state chemistry along with some serendipity contribution.

Finally, we have to return back to Fig. 1 and consider it in a reversed perspective, i.e. from the battery to new electronic and spin states and, eventually, new compounds and structures. Battery community has mastered electrochemically-driven intercalation reactions almost to perfection, turning the electrochemical cells into the chemical reactors which enable tuning magnetic, superconducting and catalytic properties^93–95^. Thus the solid-state chemists have received a powerful instrument for precise control of the chemical composition, electron/hole doping and stabilizing metastable compositions and oxidation states.

In summary, with the battery research becoming a society-driven demand, this field attracts a colossal number of researchers having wide ranges of expertise, hence enabling a multifaceted approach. Solid state chemistry should largely help to unite a vision on the many-sided problems of the discovery of novel materials and novel reactivity concepts based on combined experimental and computational methods together with the help of novel advanced characterization techniques with an improved energy and spatial resolution, preferably applied *operando*. To fulfill our prediction and hopes, such a multifaceted platform of knowledge should accelerate the development of better materials for rechargeable batteries.

## Data Availability

The raw images associated with Fig. 5d are available from the corresponding author upon reasonable request.
